# ML-CKDP: Machine learning-based chronic kidney disease prediction with smart web application

**DOI:** 10.1016/j.jpi.2024.100371

**Published:** 2024-02-22

**Authors:** Rajib Kumar Halder, Mohammed Nasir Uddin, Md. Ashraf Uddin, Sunil Aryal, Sajeeb Saha, Rakib Hossen, Sabbir Ahmed, Mohammad Abu Tareq Rony, Mosammat Farida Akter

**Affiliations:** a Dept. of Computer Science and Engineering, Jagannath University, Dhaka 1100, Bangladesh; b School of Information Technology, Deakin University, Geelong 3125, Australia; c Dept. of Cyber Security, Bangabandhu Sheikh Mujibur Rahman Digital University, Kaliakoir, Gazipur 1750, Bangladesh; d Dept. of Educational Technology, Bangabandhu Sheikh Mujibur Rahman Digital University, Kaliakoir, Gazipur 1750, Bangladesh; e Dept. of Statistics, Noakhali Science and Technology University, Noakhali 3814, Bangladesh

**Keywords:** Chronic kidney diseases, Machine learning, Feature selection, Classification

## Abstract

Chronic kidney diseases (CKDs) are a significant public health issue with potential for severe complications such as hypertension, anemia, and renal failure. Timely diagnosis is crucial for effective management. Leveraging machine learning within healthcare offers promising advancements in predictive diagnostics. In this paper, we developed a machine learning-based kidney diseases prediction (ML‐CKDP) model with dual objectives: to enhance dataset preprocessing for CKD classification and to develop a web-based application for CKD prediction. The proposed model involves a comprehensive data preprocessing protocol, converting categorical variables to numerical values, imputing missing data, and normalizing via Min-Max scaling. Feature selection is executed using a variety of techniques including Correlation, Chi-Square, Variance Threshold, Recursive Feature Elimination, Sequential Forward Selection, Lasso Regression, and Ridge Regression to refine the datasets. The model employs seven classifiers: Random Forest (RF), AdaBoost (AdaB), Gradient Boosting (GB), XgBoost (XgB), Naive Bayes (NB), Support Vector Machine (SVM), and Decision Tree (DT), to predict CKDs. The effectiveness of the models is assessed by measuring their accuracy, analyzing confusion matrix statistics, and calculating the Area Under the Curve (AUC) specifically for the classification of positive cases. Random Forest (RF) and AdaBoost (AdaB) achieve a 100% accuracy rate, evident across various validation methods including data splits of 70:30, 80:20, and K-Fold set to 10 and 15. RF and AdaB consistently reach perfect AUC scores of 100% across multiple datasets, under different splitting ratios. Moreover, Naive Bayes (NB) stands out for its efficiency, recording the lowest training and testing times across all datasets and split ratios. Additionally, we present a real-time web-based application to operationalize the model, enhancing accessibility for healthcare practitioners and stakeholders.

Web app link: https://rajib-research-kedney-diseases-prediction.onrender.com/

## Highlights

• Research introduces a multi-classifier model for CKD prediction.
• Comprehensive feature selection identifies influential features.
• Diverse dataset preprocessing facilitates robust evaluation.
• User-friendly web app offers quick CKD predictions.

## Graphical abstract

**Figure:**
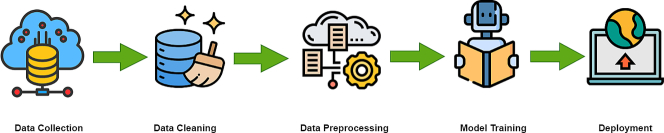

## Introduction

Chronic kidney disease (CKD) represents a significant and escalating global health challenge, increasingly recognized as a substantial contributor to morbidity and mortality worldwide. The disease's stealthy progression typically results in a delayed diagnosis, with many patients presenting at advanced stages of renal insufficiency. An alarming escalation in CKD-related deaths was highlighted by a comprehensive report covering the years 1990–2013, which documented a 90% increase in fatalities, thus ranking CKD as the 13th leading cause of death globally.^1^ This trend was further underscored by the Global Burden of Disease Study in 2010, which saw CKD rise from the 27th to the 18th position in terms of mortality.^2^ The global burden of CKD is staggering, with over 500 million individuals affected, and the disease exerts a disproportionate impact on developing regions, particularly South Asia and sub-Saharan Africa. Indeed, low- and middle-income countries are disproportionately affected, accounting for 387.5 million cases in contrast to the 110 million reported in high-income countries.^3^ A study on World Kidney Day in 2019 estimated the global impact of renal diseases to be approximately 850 million individuals and attributing to these conditions around 2.4 million deaths per year, making kidney diseases the sixth leading cause of death on the rise globally.^4^ CKD is classified into five stages based on the gradation of kidney function, with stages 1 and 2 characterized by mild, often symptomless, conditions, and stage 5 denoting terminal kidney failure.^1^ The disease prevalence in densely populated nations like Bangladesh is increasing alarmingly. Various studies have reported a 14% prevalence in six countries including Bangladesh, with urban areas such as Dhaka showing a 26% prevalence in individuals over 30 years, and other urban studies indicating a 13% prevalence in those over 15 years. In stark contrast, rural studies in Bangladesh from 2013 suggest that a third of the population is at risk of CKD, with a frequent incidence of misdiagnosis.^5^ The financial burden associated with Renal Replacement Therapy (RRT) for terminal kidney failure is prohibitively high, and in many developing countries, such treatments remain inaccessible, presenting significant obstacles to CKD management due to the scarcity of medical facilities, healthcare professionals, and the high cost of treatment. Thus, the importance of early detection of CKD cannot be overstated, as it is essential in reducing the economic impact.^1^ Traditionally, medical practitioners employ a combination of physical examinations and laboratory tests, such as blood and urine analyses, to diagnose renal diseases. These tests evaluate glomerular filtration rate (GFR) and albumin levels, respectively, to ascertain renal function and health. In the current landscape, characterized by the availability of promising data sources, the creation of robust and universally applicable diagnostic models is vital. These models support medical professionals in making accurate and timely decisions.^6^ Machine learning, an artificial intelligence branch, offers potential for early CKD detection, fostering efficient and expedient intervention. This field has become instrumental in disease diagnosis, utilizing historical patient data to predict new patient outcomes. Machine learning algorithms can assess a range of indicators, including diabetes, blood pressure, age, gender, smoking habits, creatinine levels, hypertension, and cholesterol, for CKD prognosis. The efficacy of machine learning algorithms hinges on effective feature selection. Effective feature selection streamlines machine learning by enhancing model accuracy, reducing overfitting, speeding up training, simplifying interpretation, and preventing data leakage. It's a crucial step for robust and efficient predictive modeling.^7^ In this research, we focus on addressing several key challenges that are critical for advancing the predictive capabilities in the realm of CKD. These challenges include:

1. Complexity of CKD progression: CKD often progresses without noticeable symptoms, leading to late-stage diagnoses. Our ML algorithms are designed to detect early-stage CKD by identifying subtle patterns in patient data, facilitating timely interventions.
2. Integration of diverse data sources: Diagnosing CKD requires analyzing various data types, including demographics, clinical symptoms, lab results, and comorbidities. Our research integrates these diverse data sources into a comprehensive ML model for a more accurate prediction of CKD.
3. Optimal feature selection and data preprocessing: The effectiveness of ML in health care heavily relies on the selection of relevant features. We employ advanced data preprocessing and feature selection techniques, such as correlation, chi-square, variance threshold, and others, to enhance our model's predictive accuracy.
4. Model selection and validation: Selecting the most appropriate ML model for CKD prediction is crucial. Our research compares multiple classifiers and evaluates their performance to identify the most suitable model for CKD prediction.
5. Real-time application in clinical settings: To bridge the gap between research and practical clinical application, we developed a user-friendly, web-based application. This tool facilitates real-time CKD risk assessment, aiding healthcare practitioners in making informed decisions.
6. Addressing economic and accessibility challenges: Recognizing the economic burden and accessibility issues of CKD in developing regions, our web application provides a cost-effective and accessible solution for early CKD risk assessment, especially in resource-limited settings.

By addressing these challenges, our work not only contributes to the accuracy and early detection of CKD but also aims to improve patient care and outcomes globally. This paper presents our methodology, the development of the ML model, and the design of the web application, illustrating their potential impact in the realm of CKD prediction and health care.

The remainder of this paper is organized as follows. The second section delves into existing methods and available techniques. Following that, the third section provides a detailed overview of the dataset used in this research. The fourth section outlines our proposed methodology. Moving forward, the fifth section presents the experimental results and engages in a discussion of the proposed model. In the sixth section, we describe the development of the web application, covering aspects such as the programming languages and tools utilized, the user interface design, and the application's functionality. Subsequently, the seventh section is dedicated to a comprehensive discussion of the findings. Finally, the eighth section concludes the paper by summarizing the key points and insights derived from the study.

## Literature review

This section provides a comprehensive review of the relevant literature on the prediction of CKDs using a range of machine learning and deep learning techniques. Within this review, we also highlight the limitations identified in previous works, shedding light on areas where further research and improvement are needed:

M.A. Islam et al. (2023)^8^ conducted a study on the early detection of CKD using machine learning. They worked with a dataset of 400 cases, featuring 24 attributes—13 categorical and 11 numerical. After preprocessing, Principal Component Analysis (PCA) was applied to determine key features for CKD prediction. The XgBoost classifier outperformed other algorithms, reaching an accuracy of 98.33% with the original data and improving to 99.16% after PCA was applied. Other classifiers also achieved an accuracy of 98.33% before PCA.

R. Sawhney et al. (2023)^9^ developed AI models to predict and assess CKD, using a dataset with 400 cases and 24 features, both categorical and numerical. They utilized a Multilayer Perceptron (MLP) with backpropagation, integrating two feature extraction and three feature selection techniques to improve efficiency. The Artificial Neural Network (ANN) model they created outperformed other classifiers, achieving a perfect testing accuracy of 100%, significantly higher than the Logistic Regression (LR)and Support Vector Machine (SVM) scores of 96% and 82%, respectively.

Alsekait et al. (2023)^6^ developed an ensemble deep learning model to predict CKD using a dataset of 400 cases with 24 features. The process involved data preprocessing, including label encoding and outlier detection, followed by feature selection through methods like mutual information and Recursive Feature Elimination (RFE). The model used a stacked approach combining RNN, LSTM, and GRU models, with a Support Vector Machine (SVM) for meta-learning. This model achieved high performance metrics, with an accuracy, precision, recall, and F1 score all around 99.69%.

Arif M.S. et al. (2023)^10^ designed a machine learning model to predict CKD, incorporating advanced preprocessing, feature selection using the Boruta algorithm, and hyperparameter optimization. Their method involved iterative imputation for missing values and a novel sequential data scaling technique that included robust scaling, z-standardization, and min-max scaling. The model, tested on the UCI CKD dataset with 400 cases and 24 features, achieved an accuracy rate of 100% using k-Nearest Neighbors (KNN) algorithm and grid-search CV for optimization.

Poonia RC et al. (2022)^11^ developed a feature-based model for kidney disease detection using a dataset with 400 cases and 24 features. They employed machine learning algorithms like KNN, ANN, SVM, and Naive Bayes, along with Recursive Feature Elimination (RFE)and chi-Square tests for feature selection. The model was evaluated on a dataset with healthy and diseased individuals. A logistic regression model, optimized with Chi-Square selected features, achieved the highest accuracy of 98.75%.

Pal. S. (2022)^12^ developed a model to predict CKD using a dataset of 400 instances with 24 features from the UCI machine learning repository. The study applied Logistic logistic Regression (LR), Decision Tree (DT), and Support Vector Machine (SVM) classifiers, and improved model performance with a bagging ensemble method. The Decision Tree (DT) classifier achieved the highest accuracy of 95.92%, which increased to 97.23% after implementing the bagging method.

K.M. Almustafa (2021)^13^ proposed a classification system for kidney diseases using a dataset of 400 cases with 24 features. Various machine learning classifiers were tested, with J48 and Decision Tree (DT) performing best, achieving 99% accuracy. Post-feature selection, these classifiers, along with Naive Bayes and KNN, showed improved accuracy, indicating the effectiveness of feature selection in enhancing model performance.

Ilyas et al. (2021)^14^ developed a decision tree-based diagnosis system for CKDs using a dataset with 400 cases and 24 features. They utilized J48 and Random Forest (RF) algorithms, highlighting the importance of the Glomerular Filtration Rate (GFR) calculated using the CKD-EPI equation. The model was assessed across five CKD stages, with J48 and RF performing well, particularly in early stages, with accuracies up to 98%. The performance slightly decreased in more advanced stages but remained high. Table 1 outlines the limitations identified in the previously mentioned studies.

**Table 1:** Limitations of related works.

Authors	Limitations
M.A. Islam et al. (2023)^8^	1. Limited exploration of alternative feature selection methods beyond PCA, impacting result robustness. 2. Lack of time complexity analysis hinders resource planning, scalability assessment, and real-time suitability.
R. Sawhney et al. (2023)^9^	1. Missing value handling and data scaling techniques were not applied, potentially impacting data quality and model performance. 2. The absence of time complexity analysis hinders resource allocation, real-time suitability, scalability assessment, and model selection.
Alsekait D.M. et al. (2023)^6^	1. Data scaling was not employed, potentially affecting model performance. 2. The absence of cross-validation raises concerns about model reliability and generalizability. 3. Lack of time complexity analysis hinders resource planning, real-time suitability, and scalability assessment.
Arif M.S. et al. (2023)^10^	1. Limited classifier variety (Naive Bayes and KNN) restricts performance exploration. 2. Lack of cross-validation raises reliability and generalizability concerns. 3. Absence of time complexity analysis hinders resource planning, real-time suitability, and scalability assessment.
Poonia RC et al. (2022)^11^	1. Lack of embedded feature selection may hinder optimal subset discovery. 2. Limited model and cross-validation variety may affect findings' generalizability. 3. Absence of ensemble model exploration limits model diversity. 4. Data scaling was not utilized, potentially affecting model performance. 5. Lack of time complexity analysis hampers resource planning and real-time suitability.
Pal S. (2022)^12^	1. No feature selection may cause overfitting and reduced model robustness. 2. Lack of data scaling may affect model performance. 3. Absence of time complexity analysis hampers resource planning and real-time suitability.
K.M. Almustafa (2021)^13^	1. Lack of data scaling may affect model performance. 2. Limited cross-validation settings may limit findings' generalizability. 3. Absence of time complexity analysis hampers resource planning and real-time suitability.
Ilyas et al. (2021)^14^	1. No feature selection may lead to overfitting and reduced model robustness. 2. Lack of categorical data handling and data scaling techniques may affect data quality and model performance. 3. Limited cross-validation settings and absence of time complexity analysis hinder findings' generalizability and resource planning for real-time applications.

## Dataset construction

The dataset utilized in this research was obtained from the publicly available CKD collection hosted on the UCI Machine Learning Repository.^15^ This dataset, with a file size of 43 KB, is specifically tailored for research in the field of medical diagnosis, particularly focusing on CKD. This dataset, donated on July 2, 2015, by L. Rubini, P. Soundarapandian, and P. Eswaran, is specifically designed for predicting CKD and was collected over approximately 2 months from a hospital. The dataset is characterized by the following features:

a. Multivariate nature: It includes various types of data.
b. Associated tasks: Primarily for classification tasks.
c. Feature types: Real-valued features.
d. Number of instances: 400.
e. Number of features: 25, including demographic, clinical, and laboratory data.

The dataset contains 24 features plus a class attribute, with a mix of 11 numerical and 14 nominal variables. The class label indicates the presence or absence of CKD; '1' denotes a positive CKD diagnosis, while '0' signifies a negative diagnosis. Presence of missing data can significantly impact the performance and accuracy of machine learning models. The dataset seems to have missing values (NaN) in several columns. Handling these missing values through techniques like imputation or deletion will be crucial. Columns like rbc, pc, pcc, ba, htn, dm, cad, appet, pe, ane, and classification appear to be categorical. Depending on the machine learning algorithm used, these may need to be encoded into numerical values. A detailed breakdown of the categorical and numeric attributes is provided in Table 2.

**Table 2:** Dataset description.

#	Column Name	Attribute	Data Type	Range	Description
1	Age	Age	Numerical	(2–90)	Patient’s age in years
2	Blood Pressure	BP	Numerical	(50–180)	Patient’s blood pressure in mmHG
3	Specific gravity	SG	Categorical	(1.005, 1.010, 1.015, 1.020, 1.025)	The ratio between urine density to water density
4	Albumin	AL	Categorical	(0, 1, 2, 3, 4, 5)	Protein percentage in blood plasma
5	Sugar	SU	Categorical	(0, 1, 2, 3, 4, 5)	The sugar level in blood plasma
6	Red blood cells	RBC	Categorical	(Abnormal, Normal)	Percentage of red blood cells in blood plasma
7	Pus cell	PC	Categorical	(Abnormal, Normal)	White blood cells in urine
8	Pus cell clumps	PCC	Categorical	(Abnormal, Normal)	Sign of bacterial infection
9	Bacteria	BA	Categorical	(Present, Not present)	Sign of bacterial existence in urine
10	Blood glucose random	BGR	Numerical	(22–490)	A random test of glucose in the blood in mg/dL
11	Blood urea	BU	Numerical	(1.5–391)	Percentage of urea nitrogen in blood plasma
12	Serum creatine	SC	Numerical	(0.4–76)	Creatine level in patient muscles in mg/dL
13	Sodium	SOD	Numerical	(4.5–163)	Sodium mineral level in blood
14	Potassium	POT	Numerical	(2.5–47)	Potassium mineral level in blood
15	Hemoglobin	HEMO	Numerical	(3.1–17.8)	Red protein that responsible of transport oxygen in the blood
16	Packed cell volume	PCV	Numerical	(9–54)	The volume of blood cells in a blood sample
17	White blood cell count	WC	Numerical	(2200–26,400)	Count of white blood cells in cells/cumm
18	Red blood cell count	RC	Numerical	(2.1–8)	Count of red blood cells in millions/cumm
19	Hypertension	HTN	Categorical	(Yes, No)	The condition where there is continuously high pressure in the blood vessels
20	Diabetes mellitus	DM	Categorical	(Yes, No)	Impairment in the body’s production or response to insulin, a condition of glucose metabolism that makes it difficult to maintain healthy levels of sugar
21	Coronary artery diseases	CAD	Categorical	(Yes, No)	A common heart condition where the main blood channels feeding the heart, have trouble supplying enough nutrients, oxygen, and blood to the heart muscle
22	Appetite	APPET	Categorical	(Good, Poor)	The desire to eat food
23	Pedal edema	PE	Categorical	(Yes, No)	Swelling of the patient’s body due to an injury or inflammation
24	Anemia	ANE	Categorical	(Yes, No)	Insufficient healthy red blood cells to transport appropriate oxygen to the body’s tissues
25	Class	Class	Categorical	(CKD, Not CKD)	A positive or negative result in terms of having chronic kidney diseases

## Methodology

The presented methodology constitutes a structured and systematic approach designed to preprocess and optimize a dataset, subsequently facilitating its suitability for classification tasks. The primary aim is to augment the dataset's appropriateness for classification by employing a comprehensive preprocessing pipeline. We developed seven preprocessed datasets and conducted an evaluation of the error rates for nine different classifiers on each dataset, as detailed in Table 3. Subsequently, we identified the top seven classifiers: Random Forest (RF), AdaBoost (AdaB), Gradient Boosting (GB), XgBoost (XgB), Naive Bayes (NB), Support Vector Machine (SVM), and Decision Tree (DT). These were chosen for their consistently low error rates across the majority of the datasets, as demonstrated in the table.

**Table 3:** Classifier error rates.

	RF	XgB	GB	AdaB	LR	SVM	DT	NB	KNN
Dataset_1	0.0125	0.0175	0.0124	0.0150	0.0375	0.0275	0.0300	0.0300	0.3753
Dataset_2	0.0100	0.0150	0.0200	0.0050	0.0350	0.0225	0.0250	0.0300	0.3751
Dataset_3	0.0725	0.0775	0.0725	0.0725	0.0725	0.0725	0.0725	0.0725	0.3750
Dataset_4	0.0125	0.0125	0.0124	0.0225	0.0575	0.0250	0.0349	0.0325	0.0275
Dataset_5	0.0150	0.0200	0.0150	0.0175	0.0450	0.0225	0.0250	0.0325	0.0225
Dataset_6	0.1801	0.1801	0.1801	0.1801	0.1801	0.1801	0.1801	0.1801	0.3750
Dataset_7	0.0375	0.0474	0.0450	0.0475	0.0375	0.0600	0.0525	0.0750	0.0450

Phase 1 of the methodology involves a series of preprocessing steps directed at enhancing the quality and relevance of the initial dataset for classification purposes. The process commences with the transformation of categorical variables into numerical representations through label encoding, rendering them suitable for analysis. Following this, missing values are imputed using the mean value to mitigate the impact of data incompleteness. Subsequently, Min-Max scaling is employed to scale each data point within the range of 0 to 1, ensuring uniformity across numeric features. In Phase 2, three distinct feature selection methods are utilized to create data subsets, each emphasizing different facets of feature relevance. Feature selection encompasses three main types: filter, wrapper, and embedded methods.^7^ Filter methods assess individual features based on statistical metrics like correlation or mutual information, ranking them independently before applying a threshold to select the most informative ones. While computationally efficient, filter methods may overlook feature interactions.^16^, ^17^, ^18^, ^19^ Wrapper methods, on the other hand, evaluate feature subsets by training and evaluating models with different feature combinations, employing search strategies like forward selection or backward elimination to find the optimal subset. While effective, wrapper methods can be computationally expensive due to repeated model training.^20^, ^21^, ^22^, ^23^ Embedded methods seamlessly integrate feature selection into the model training process, selecting features based on their relevance to model performance. Techniques like Lasso regression and decision trees employ embedded feature selection, offering computational efficiency well-suited for larger datasets.^24^, ^25^, ^26^ In our proposed methodology we have used seven feature selection algorithms. Using multiple feature selection techniques in model development is highly effective because it leverages the unique strengths of each method to create a more robust and accurate predictive model. This approach ensures a comprehensive analysis of features, reducing overfitting, effectively handling multicollinearity, and optimizing the selection of the most relevant features. Consequently, it leads to improved model accuracy and interpretability, while also being resource-efficient. This diversified approach is key to adapting the model to the specific nuances of the dataset, enhancing its overall performance and reliability.

In the first approach, the preprocessed dataset is subjected to a heatmap analysis to uncover correlations between features, allowing for dimensionality reduction and the selection of relevant features. It helps in reducing redundancy in the model. This not only simplifies the model but can also improve performance by focusing on the most relevant features and avoiding the noise introduced by redundant data. This results in preprocessed Dataset_1. In the second approach, the Chi-Square statistical test is applied for feature selection, highlighting relationships between categorical variables and bolstering feature relevance. By evaluating the independence of each feature with respect to the target variable, it helps in selecting features that have a significant association with the outcome. This enhances the model's ability to distinguish between different classes, thus improving classification accuracy. This results in the creation of preprocessed Dataset_2. In the third approach, a variance threshold technique is employed to filter out features with low variance, leading to the creation of preprocessed Dataset_3. Features with low variance often do not contribute much to the model's predictive power, so their removal can make the model more efficient and potentially more accurate. The fourth approach involves Recursive Feature Elimination (RFE), which iteratively removes less significant features, yielding preprocessed Dataset_4. RFE ensures that the final model is not burdened with irrelevant or less important features, thereby improving model interpretability and potentially its predictive performance. In the fifth approach, sequential forward selection (SFS) is utilized to progressively add features based on their contribution to predictive accuracy, generating preprocessed Dataset_5. This method is particularly useful for identifying the optimal combination of features, enhancing the model's predictive accuracy without overfitting. Subsequently, two variations of feature selection are conducted. In the sixth approach, Lasso regression is employed to select relevant features while penalizing less significant ones, producing preprocessed Dataset_6. By penalizing the coefficients of less significant features, it effectively reduces overfitting and enhances model generalizability. This results in a more robust model that performs well on unseen data. In the seventh approach, Ridge Regression is utilized to address multicollinearity and accentuate essential features, resulting in preprocessed Dataset_7. By adding a degree of bias to the regression estimates, it stabilizes the estimates of correlated features. This leads to more reliable predictions, especially in cases where predictor variables are highly correlated. Table 4 displays the features selected by various feature selection algorithms.

**Table 4:** Selected features by different features selection algorithms.

Correlation	Chi square	Variance threshold	RFE	SFS	Lasso	Ridge regression
AGE, BP, SG, AL, SU, BGR, BU, SC, SOD, POT, HEMO, WC, RBC, PC, PCC, BA, HTN, DM, CAD, APPET, PE, ANE	SG, AL, SU, BGR, BU, SC, HEMO, PCV, RC, PC, PCC, BA, HTN, DM, CAD, APPET, PE, ANE	RBC, PC, HTN, DM, APPET, PE, ANE	SG, AL, BGR, BU, SC, SOD, HEMO, PCV, RC, HTN, DM	BP, SG, AL, BGR, BU, SC, HEMO, HTN, DM, APPET, ANE	HTN, DM	BP, AL, BGR, SC, POT, WC, PC, HTN, DM, APPET, PE

**Fig. 1:**
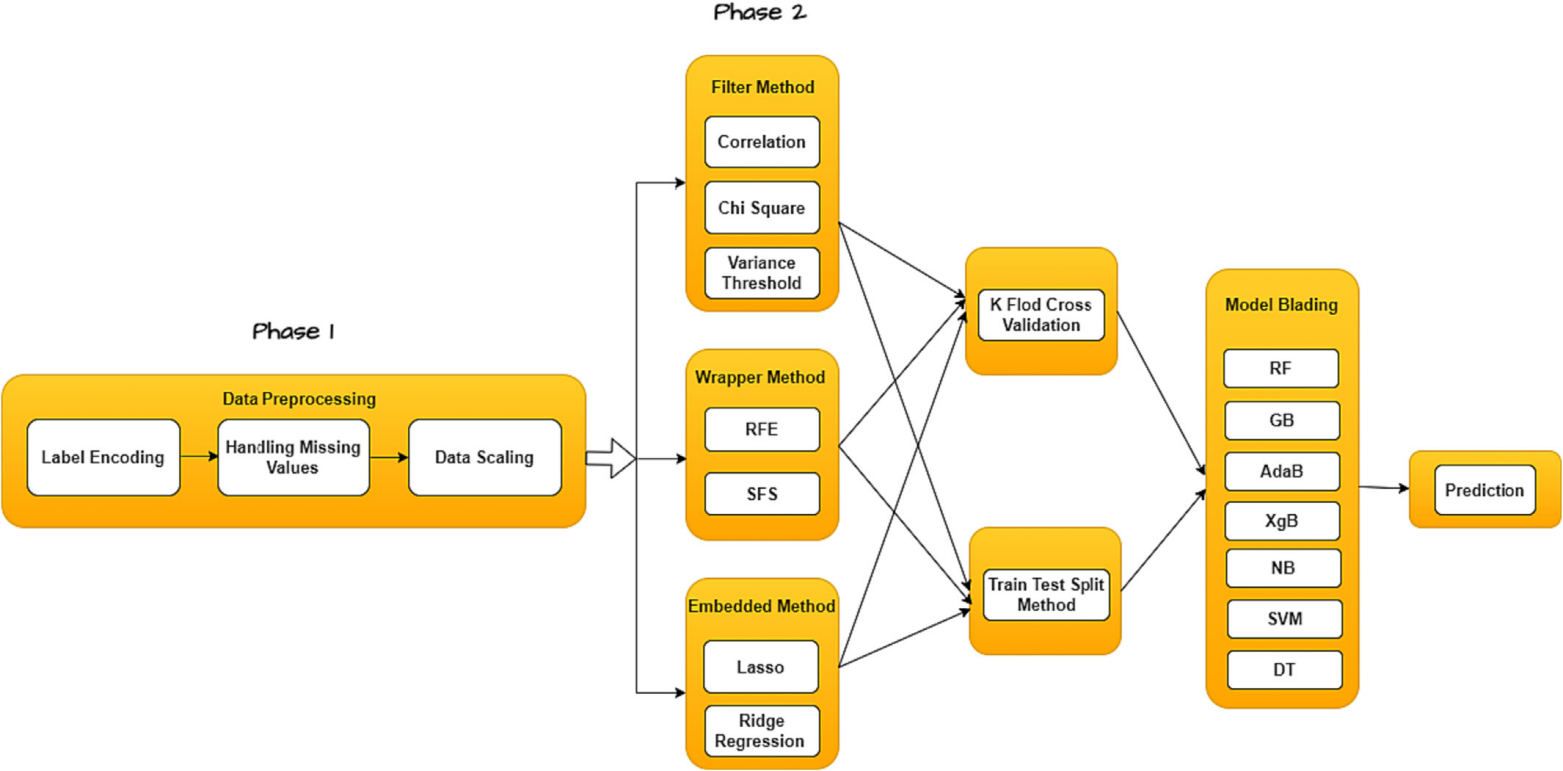
Proposed methodology.

Each of these preprocessed datasets is tailored to emphasize the strengths of their respective feature selection methods. Following the thorough preprocessing and feature selection phases, the methodology proceeds to the classification stage. Seven different classifiers, namely Random Forest (RF), AdaBoost (AdaB) Gradient Boosting (GB), XgBoost (XgB), Naive Bayes (NB), Support Vector Machine (SVM), and Decision Tree (DT), are employed on each preprocessed dataset. This approach ensures a rigorous assessment of their performance and helps identify the most suitable dataset and classifier combination for the classification task. The performance of each classifier is assessed using two distinct evaluation strategies: the train-test splitting method, which provides insights into model generalization, and stratified k-fold cross-validation, offering robust performance estimates. The above-mentioned steps are illustrated in Fig. 1. This methodology offers several advantages. Firstly, it ensures comprehensive dataset preprocessing, resulting in improved data quality and noise reduction. Secondly, it leverages various feature selection methods, allowing for dataset customization to align with the strengths of each method and potentially uncover hidden patterns. Lastly, the evaluation of multiple classifiers using both train-test splitting and cross-validation guarantees a thorough evaluation of model performance and robustness. Ultimately, the objective of this methodology is to identify the most suitable dataset and classifier combination for the specific classification task, thereby providing valuable insights for subsequent analysis or model deployment.

## Result analysis

The performance of a machine learning model is evaluated using a performance metrics. The matrix module from the “scikit-learn” library offers essential functions for computing various performance evaluation metrics. Among the evaluation tools is the confusion matrix, which provides four outcomes based on the datasets: True Positive (TP), True Negative (TN), False Positive (FP), and False Negative (FN). These outcomes are instrumental in calculating key metrics such as accuracy, precision, True Positive Rate (TPR), False Positive Rate (FPR), True Negative Rate (TNR), and False Negative Rate (FNR) through the following equations^31^:

(1) $$\text{Accuracy}=\frac{\mathrm{TN}+\mathrm{TP}}{\mathrm{TN}+\mathrm{TP}+\mathrm{FN}+\mathrm{FP}}$$

(2) $$\text{Precision}=\frac{\mathrm{TP}}{\mathrm{TP}+\mathrm{FP}}$$

(3) $$\text{Sensitivity}/\text{True Positive Rarte}/\text{Recall}=\frac{\mathrm{TP}}{\mathrm{TP}+\mathrm{FN}}$$

(4) $$\text{False Positive Rate}=\frac{\mathrm{FP}}{\mathrm{FP}+\mathrm{TN}}$$

(5) $$\text{Specificity}/\text{True Negative Rate}=\frac{\mathrm{TN}}{\mathrm{TN}+\mathrm{FP}}$$

(6) False Negative Rate=1−TPR

**Fig. 2:**
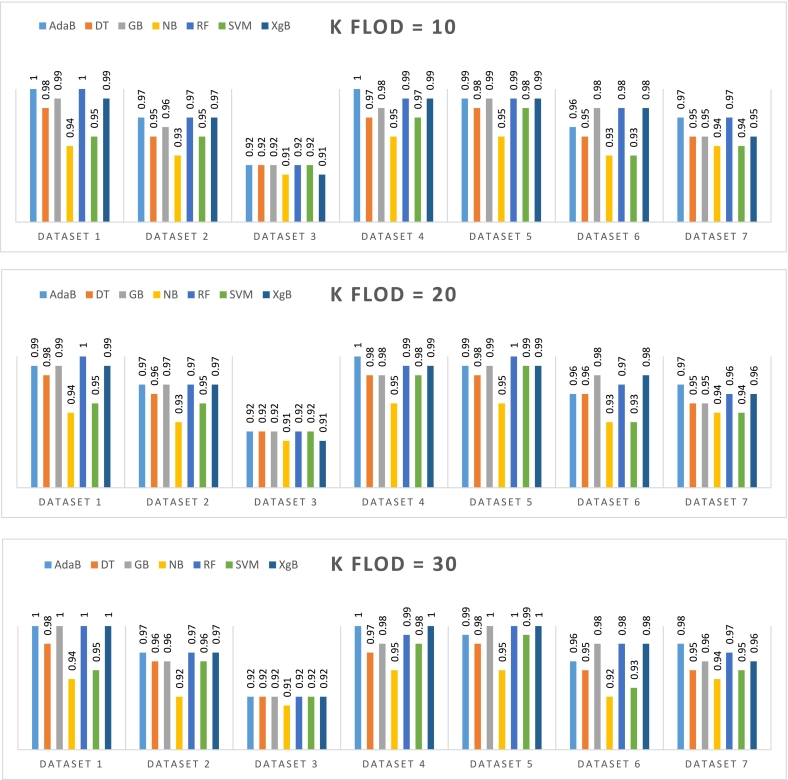
Accuracy of different classifiers for different splitting ratios based on k-fold cross-validation.

In this study, a sophisticated multi-pipeline approach was implemented on the CKD dataset using Python as the programming language. The objective was to apply advanced feature selection techniques to distill a subset of significant features that could enhance the predictive performance of various machine learning models. The feature selection process was followed by the deployment of an array of classifiers, including RF, GB, AdaB, XgB, NB, Support SVM, and DT. These classifiers were utilized through the capabilities provided by the scikit-learn library, a powerful tool in the Python ecosystem for machine learning tasks. For the purpose of classification, the dataset was subjected to two distinct data splitting methodologies: the train-test split and the k-fold cross-validation. These methods are critical in validating the generalizability and robustness of the classifiers. The accuracy of these classifiers was then meticulously computed and compared, providing insight into each model's efficiency in accurately classifying instances as either indicative of CKD or as non-CKD. The careful analysis of each classifier's outcomes facilitated an in-depth understanding of the feature sets that yielded the highest classification accuracy. This comprehensive examination was instrumental in discerning the most effective machine learning strategies for distinguishing between the two health states represented within the CKD dataset. Such a rigorous approach ensures that the models selected for potential clinical support applications are both reliable and valid. Fig. 2 depicts the accuracy metrics for various classifiers across different k-fold cross-validation splitting ratios.

In Fig. 2 when the k-fold cross-validation is set to 10, the RF and AdaB classifiers emerge as the top performers, showcasing robust accuracy across the board. RF even achieves a perfect score on Dataset_4. In contrast, SVM and NB lag behind, with notably lower accuracy figures. The highest accuracies are recorded on Dataset_4 for RF and AdaB, while the most challenging dataset for classifiers appears to be Dataset_3. With the K-fold increased to 20, the RF classifier continues to excel, maintaining perfect accuracy on Dataset_4 and high performance elsewhere. Other classifiers like GB, AdaB, and XgB also show enhanced accuracy, particularly in Datasets 1, 4, 5, and 6. Yet again, SVM and NB do not measure up to their counterparts. At a k-fold of 30, there's a marked performance spike for RF, AdaB, GB, and XgB, with RF and XgB hitting the mark with perfect scores in Datasets 1 and 4. SVM sees a marginal accuracy improvement in Dataset_2 at this higher k-fold setting, whereas NB continues to underperform relative to other classifiers. In summary, the dataset reveals that RF is the most consistent high achiever across various k-fold settings, with AdaB, GB, and XGB also demonstrating strong performance. The SVM and Naive Bayes classifiers appear to be less effective. Although Dataset_3 poses the greatest challenge across all classifiers, Dataset_4 is where they tend to excel. Increasing the k-fold value tends to benefit accuracy but not significantly so, hinting at a nuanced relationship between k-fold values and classifier performance.

The Area Under the Curve (AUC) is a pivotal metric in assessing classification models, serving an integral role in both machine learning and statistical analyses. This metric evaluates the model's proficiency in distinguishing between the positive and negative classes over a range of classification thresholds. Its prominence in analysis is attributed to several distinct advantages. Firstly, AUC offers a condensed, yet comprehensive view of a model’s performance, encapsulating the balance between the TPR (sensitivity) and FPR (1-specificity). This solitary metric becomes instrumental in gauging the model’s capability to discriminate between classes. Furthermore, the AUC curve is instrumental in pinpointing the most suitable classification threshold, a vital aspect in situations where distinct costs are associated with FP and FN. In addition to these, the AUC enables a straightforward comparison between disparate models or algorithms. A higher AUC value signifies enhanced performance, offering a clear, quantitative measure for evaluation. The AUC metric is notably resilient, holding its ground especially when faced with class imbalances, underscoring its reliability in diverse settings. The visual rendition of the AUC curve serves to delineate the model’s responses across varying thresholds, granting insights into its adaptability and performance in assorted conditions. Consequently, the AUC is indispensable in offering a holistic view of the classification model’s efficacy, facilitating informed decisions on threshold selection, and providing a robust platform for comparing diverse models. It stands as a bulwark, ensuring rigorous evaluations across a spectrum of domains.^27^, ^28^, ^29^, ^30^, ^32^, ^33^ Table 5 delineates the AUC values associated with different classifiers, analyzed over varied splitting ratios and founded upon k-fold cross-validation. This tabulation aids in an in-depth appraisal, anchoring the comparative analysis of model performance.

**Table 5:** AUC values of different classifiers for different splitting ratios based on k-fold cross-validation.

Datasets	K-Fold	AdaB	DT	GB	NB	RF	SVM	XgB
Dataset_1	10	1.00	0.98	1.00	0.99	1.00	1.00	1.00
	20	1.00	0.98	1.00	0.99	1.00	1.00	1.00
	30	1.00	0.98	1.00	0.98	1.00	1.00	1.00
Dataset_2	10	0.99	0.98	0.99	0.98	0.99	0.99	0.99
	20	0.99	0.99	0.99	0.98	0.99	0.99	0.99
	30	0.99	0.98	0.99	0.98	0.99	0.99	0.99
Dataset_3	10	0.94	0.94	0.94	0.93	0.94	0.94	0.94
	20	0.94	0.94	0.94	0.93	0.94	0.94	0.94
	30	0.94	0.94	0.94	0.93	0.94	0.94	0.94
Dataset_4	10	1.00	0.98	1.00	0.99	1.00	1.00	1.00
	20	1.00	0.98	1.00	0.99	1.00	1.00	1.00
	30	1.00	0.98	1.00	0.99	1.00	1.00	1.00
Dataset_5	10	1.00	0.98	1.00	0.99	1.00	1.00	1.00
	20	1.00	0.98	1.00	0.99	1.00	1.00	1.00
	30	1.00	0.98	1.00	0.99	1.00	1.00	1.00
Dataset_6	10	0.99	0.95	1.00	0.98	1.00	0.98	1.00
	20	0.99	0.95	1.00	0.98	1.00	0.98	1.00
	30	1.00	0.95	1.00	0.98	1.00	0.98	1.00
Dataset_7	10	1.00	0.95	0.99	0.98	1.00	0.99	0.99
	20	1.00	0.95	0.99	0.98	0.99	0.99	0.99
	30	0.99	0.96	0.99	0.97	1.00	0.99	0.99

In Table 5**,** AdaB stands out for its consistency, delivering perfect or near-perfect AUC values of 1.00 across nearly all datasets and partitions. Its performance only slightly dips in Dataset_3 with the 10, 20, and 30-fold partitions, where the AUC values fall to 0.94, indicating a potential area of weakness. The DT classifier also shows high efficacy, typically around 0.98 AUC, but this is compromised in Dataset_6 across all k-fold variations and again in Dataset_7 when the partition increases to 30-fold. GB is noteworthy for its stability, maintaining high AUC values across all datasets and k-fold partitions, suggesting a robustness to partition size. In contrast, NB indicates some sensitivity to the cross-validation strategy, with a minor decrease in AUC values in Dataset_3 across all partitions and a more noticeable drop to 0.97 in Dataset_7 with the 30-fold partition. RF mirrors the strong performance of AdaB, achieving a consistent 1.00 AUC across almost all datasets and partitions, reinforcing its reliability across different cross-validation scenarios. The SVM, while generally strong, shows a slight decrease to 0.98 AUC in Dataset_6 across all k-fold partitions, highlighting a potential vulnerability in more challenging datasets. Finally, XGB's performance is exemplary, with perfect AUC scores in all datasets and partitions, with a single exception in Dataset_7 at the 30-fold partition where it experiences a marginal drop to 0.99 AUC. Overall, while all classifiers perform well, AdaB, GB, RF, and XgB demonstrate particularly robust performances across various k-fold partitions, suggesting they are less influenced by the number of folds in the data partitioning. DT and NB, although generally high-performing, exhibit some fluctuations with different k-fold numbers, and the SVM, while strong, shows minor inconsistencies in specific datasets.

Classifiers like AdaB, GB, RF, and XgB excel across most datasets and k-fold partitions, often achieving perfect AUC scores. This consistent performance is attributed to their robust algorithms capable of effectively handling a variety of data distributions and complexities. Their adaptability and resilience to different cross-validation strategies play a crucial role in their success. However, certain classifiers display weaknesses under specific conditions. DT's performance dips in Dataset_6 and Dataset_7 at higher k-fold partitions, possibly due to its tendency to overfit complex or noisy data. Naive Bayes shows a decrease in performance in Dataset_3 and a significant drop in Dataset_7 with the 30-fold partition, likely because of its assumption of feature independence which might not hold true in these datasets. SVM's minor inconsistencies in Dataset_6 could be due to its sensitivity to the feature space's dimensionality and distribution. The narrative culminates in Table 6, which unveils the time complexity analysis, dissected across diverse classifiers and splitting ratios and arrayed separately for training and test data, offering a granular perspective of the computational dynamics inherent in each classification paradigm.

**Table 6:** Time complexity analysis of different classifiers for different splitting ratios based on k-fold cross-validation.

		Training time (s)							Testing time (s)						
Datasets	K-Fold	AdaB	DT	GB	NB	RF	SVM	XgB	AdaB	DT	GB	NB	RF	SVM	XgB
Dataset_1	10	0.02	0.0	0.0	0.0	0.02	0.0	0.01	0.15	0.01	0.31	0.0	0.22	0.02	0.08
	20	0.02	0.0	0.0	0.0	0.02	0.0	0.01	0.18	0.01	0.34	0.0	0.29	0.03	0.09
	30	0.02	0.0	0.0	0.0	0.02	0.0	0.01	0.16	0.01	0.4	0.0	0.23	0.02	0.08
Dataset_2	10	0.02	0.0	0.0	0.0	0.02	0.0	0.01	0.14	0.0	0.16	0.0	0.23	0.02	0.08
	20	0.01	0.0	0.0	0.0	0.02	0.0	0.01	0.12	0.0	0.18	0.0	0.26	0.02	0.08
	30	0.02	0.0	0.0	0.0	0.02	0.0	0.0	0.14	0.0	0.15	0.0	0.25	0.02	0.09
Dataset_3	10	0.02	0.0	0.0	0.0	0.02	0.0	0.0	0.13	0.0	0.13	0.0	0.24	0.01	0.06
	20	0.02	0.0	0.0	0.0	0.02	0.0	0.0	0.14	0.0	0.17	0.0	0.2	0.02	0.06
	30	0.01	0.0	0.0	0.0	0.02	0.0	0.0	0.12	0.0	0.15	0.0	0.22	0.02	0.06
Dataset_4	10	0.02	0.0	0.0	0.0	0.02	0.0	0.01	0.17	0.01	0.4	0.0	0.32	0.02	0.08
	20	0.01	0.0	0.0	0.0	0.02	0.0	0.01	0.15	0.01	0.38	0.0	0.23	0.02	0.07
	30	0.02	0.0	0.0	0.0	0.02	0.0	0.01	0.17	0.01	0.33	0.0	0.26	0.02	0.07
Dataset_5	10	0.02	0.0	0.0	0.0	0.02	0.0	0.0	0.17	0.01	0.2	0.0	0.2	0.02	0.07
	20	0.01	0.0	0.0	0.0	0.02	0.0	0.0	0.12	0.0	0.2	0.0	0.23	0.02	0.07
	30	0.02	0.0	0.0	0.0	0.02	0.0	0.0	0.16	0.0	0.21	0.0	0.22	0.01	0.06
Dataset_6	10	0.02	0.0	0.0	0.0	0.02	0.0	0.0	0.17	0.01	0.26	0.0	0.22	0.03	0.08
	20	0.02	0.0	0.0	0.0	0.02	0.0	0.01	0.17	0.0	0.26	0.0	0.25	0.03	0.09
	30	0.01	0.0	0.0	0.0	0.02	0.0	0.01	0.14	0.01	0.3	0.0	0.22	0.02	0.08
Dataset_7	10	0.01	0.0	0.0	0.0	0.02	0.0	0.01	0.13	0.01	0.24	0.0	0.28	0.02	0.08
	20	0.02	0.0	0.0	0.0	0.02	0.0	0.01	0.15	0.0	0.25	0.0	0.22	0.02	0.08
	30	0.01	0.0	0.0	0.0	0.02	0.0	0.0	0.13	0.01	0.23	0.0	0.24	0.02	0.08

**Fig. 3:**
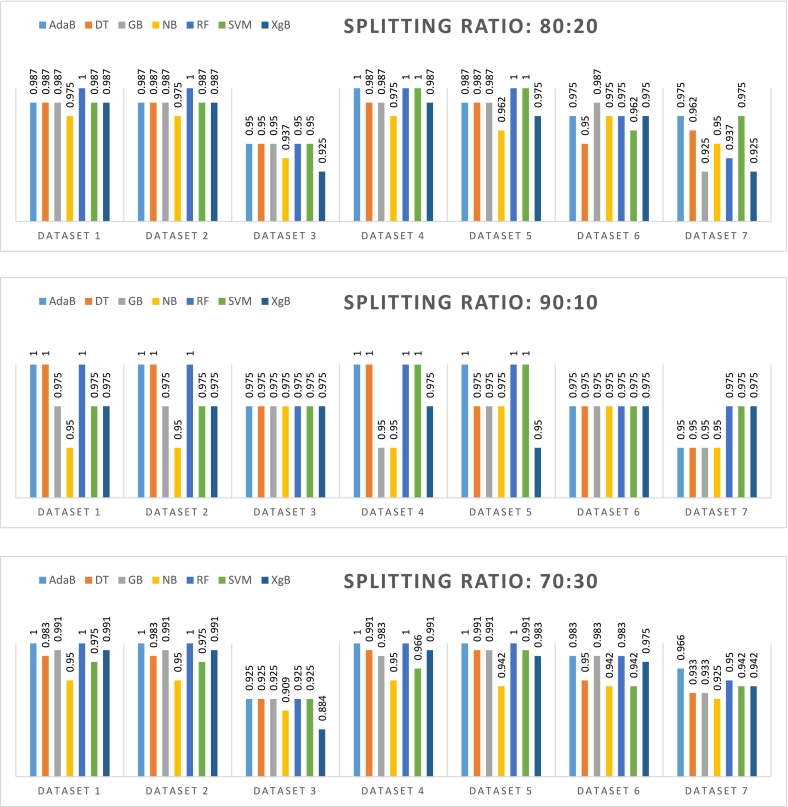
Accuracy of different classifiers for different splitting ratios based on train-test split method.

The analysis of time complexity in Table 6 reveals that for Dataset_1, classifiers like NB, DT, and SVM demonstrate extremely low training times across all k-folds, often negligible, while XgB tends to have the longest testing times, peaking at 0.31 s for 10-fold and 0.4 s for 30-fold. Moving to Dataset_2, the pattern remains with XgB having the highest testing time, particularly at 0.26 s for 20-fold. In Dataset_3, even though the training times remain low, the testing times for XgB are notably less than in the previous datasets, with the highest being 0.24 s for 10-fold. As we look at Dataset_4, there's a slight increase in the testing times for XgB, resembling the trend observed in Dataset_1, where it reached up to 0.4 s for 10-fold. Dataset_5 shows a reduction in XgB's testing times compared to Dataset_4, with the maximum time being 0.21 s for 30-fold. With Dataset_6, the testing times for XgB were consistently higher than its training times, with a peak at 0.26 s for both 10 and 20-fold. Lastly, Dataset_7 displays a similar trend to Dataset_6, with XgB's highest testing time at 0.28 s for 10-fold. Throughout the datasets, while RF and AdaB maintain consistent training times, and DT, NB, and SVM generally require the least amount of time. GB's time requirements increase with the number of k-folds, and XgB consistently has the longest testing times across all datasets, more so with an increase in the number of k-folds. This comprehensive analysis underscores how both the choice of classifier and the k-fold parameter significantly influence time complexity during cross-validation. Fig. 3 illustrates the accuracy of various classifiers at differing train-test split ratios, providing a comparative analysis of model performance within the train-test split paradigm. This figure effectively encapsulates the range of outcomes observed when applying different splitting strategies to assess classifier robustness.

In Fig. 3**,** with a 90:10 train-test split, AdaB and DT both achieved perfect accuracy of 1.00 across Datasets 1, 2, 4, and 5, with a slight decrease on Datasets 6 and 7. GB and NB maintained accuracies within the range of 0.95–0.97 for all datasets. RF excelled, hitting perfect accuracies on Datasets 1, 2, 4, 5, and 7, and slightly lower on Datasets 3 and 6. SVM varied from 0.95 to 0.97, and XgB ranged from 0.92 on Dataset_3 to 0.98 on Dataset_4. At an 80:20 ratio, AdaB's accuracy ranged from 0.95 on Dataset_3 to a perfect score on Dataset_4. DT improved slightly on some datasets with accuracies between 0.95 and 0.98. GB's performance showed consistency with the 90:10 split, with a slight dip to 0.92 on Dataset 7. NB showed a minor decrease, scoring between 0.93 on Dataset 3 to 0.97 on Datasets 2, 4, and 5. RF performed robustly, with a perfect score on Dataset_4 and the lowest at 0.93 on Dataset 7. SVM scored between 0.95 and 1.00, showing improvement on Dataset_4. XgB remained consistent with scores mostly between 0.92 and 0.98. With a 70:30 split, AdaB performed well, scoring from 0.92 on Dataset 3 to 1.00 on Datasets 1, 2, 4, and 5. DT ranged from 0.92 on Dataset 3 to 0.99 on Datasets 4 and 5. GB had a strong performance, with a minimum of 0.92 and a high of 0.99 on Datasets 1 and 2. NB presented accuracies from 0.93 to 0.97 across the datasets. RF showed high performance with a range from 0.93 on Dataset_7 to 1.00 on Datasets 1, 4, and 5. SVM had a slight decrease in performance with accuracies between 0.95 and 0.97. XgB displayed consistent results with scores mainly in the range of 0.92 to 0.971 . In summary, RF and AdaB are the top classifiers, showing outstanding performance across all datasets and split ratios. GB, DT, and XgB also demonstrated strong accuracies, but with some variability. NB and SVM, despite being generally effective, did not perform at the same level as the top classifiers. Further insights are gleaned from a confusion matrix analysis for these classifiers at varying split ratios, with the detailed results encapsulated in Table 7.

**Table 7:** Confusion matrix outcomes of different classifiers for different splitting ratios based on train-test split method.

Datasets	Classifiers	70:30				80:20				90:10			
		TP	FN	TN	FP	TP	FN	TN	FP	TP	FN	TN	FP
Dataset_1	RF	77	0	44	0	52	0	28	0	26	0	14	0
	GB	76	1	44	0	51	1	28	0	25	1	14	0
	AdaB	77	0	44	0	51	1	28	0	26	0	14	0
	NB	73	4	42	2	51	1	27	1	25	1	13	1
	SVM	75	2	43	1	52	0	27	1	26	0	13	1
	XgB	76	1	44	0	51	1	28	0	25	1	14	0
	DT	75	2	44	0	51	1	28	0	26	0	14	0
Dataset_2	RF	76	1	44	0	52	0	28	0	26	0	14	0
	GB	77	0	44	0	52	0	28	0	26	0	14	0
	AdaB	76	1	44	0	51	1	28	0	25	1	14	0
	NB	72	5	42	2	50	2	27	1	25	1	13	1
	SVM	72	5	44	0	50	2	28	0	25	1	14	0
	XgB	77	0	44	0	52	0	28	0	26	0	14	0
	DT	75	2	44	0	50	2	28	0	25	1	14	0
Dataset_3	RF	68	9	44	0	48	4	28	0	25	1	14	0
	GB	68	9	44	0	48	4	28	0	25	1	14	0
	AdaB	68	9	44	0	48	4	28	0	25	1	14	0
	NB	68	9	42	2	48	4	27	1	25	1	14	0
	SVM	68	9	44	0	48	4	28	0	25	1	14	0
	XgB	67	10	40	4	49	3	25	3	25	1	14	0
	DT	68	9	44	0	48	4	28	0	25	1	14	0
Dataset_4	RF	77	0	44	0	52	0	28	0	26	0	14	0
	GB	76	1	43	1	51	1	28	0	25	1	13	1
	AdaB	77	0	44	0	52	0	28	0	26	0	14	0
	NB	73	4	42	2	51	1	27	1	25	1	13	1
	SVM	73	4	44	0	52	0	28	0	26	0	14	0
	XgB	76	1	44	0	51	1	28	0	25	1	14	0
	DT	76	1	44	76	51	1	28	0	26	0	14	0
Dataset_5	RF	77	0	44	0	52	0	28	0	26	0	14	0
	GB	76	1	44	0	51	1	28	0	25	1	14	0
	AdaB	77	0	44	0	51	1	28	0	26	0	14	0
	NB	72	5	42	2	50	2	27	1	26	0	13	1
	SVM	76	1	44	0	52	0	28	0	26	0	14	0
	XgB	76	1	43	1	51	1	27	1	25	1	13	1
	DT	76	1	44	0	51	1	28	0	26	0	13	1
Dataset_6	RF	76	1	43	1	51	1	27	1	25	1	14	0
	GB	76	1	43	1	52	0	27	1	25	1	14	0
	AdaB	75	2	44	0	51	1	27	1	25	1	14	0
	NB	72	5	42	2	51	1	27	1	25	1	14	0
	SVM	70	7	44	0	49	3	28	0	25	1	14	0
	XgB	75	2	43	1	51	1	27	1	25	1	14	0
	DT	74	3	41	3	49	3	27	1	24	2	14	0
Dataset_7	RF	72	5	43	1	48	4	27	1	25	1	14	0
	GB	71	6	42	2	47	5	27	1	24	2	14	0
	AdaB	75	2	42	2	50	2	28	0	24	2	14	0
	NB	72	5	40	4	51	1	25	3	25	1	13	1
	SVM	70	7	44	0	50	2	28	0	25	1	14	0
	XgB	72	5	42	2	48	4	26	2	25	1	14	0
	DT	72	5	41	3	50	2	27	1	24	2	14	0

Table 7 displays the confusion matrix results for classifiers over varied split ratios: 70:30, 80:20, and 90:10, encompassing TP, FN, TN, and FP values. RF stands out, registering 77 TP and zero FN and FP, reflecting its adeptness in identifying positive cases. AdaB, praised for accuracy in Fig. 3, also exhibits strong confusion matrix outcomes, effectively balancing TP and TN and minimizing critical errors, marking it and RF as optimal for precision-sensitive applications like medical diagnoses. Conversely, DT and XgB displayed challenges; DT incurred 76 FP for Dataset_4, and XgB marked 10 FN for Dataset_3. These metrics, integral for evaluating classifier reliability and precision, further unfold in Table 8, where TPR, FNR, TNR, and FPR are scrutinized across classifiers and split ratios.

**Table 8:** Performance evaluation matrices of different classifiers for different splitting ratios based on train-test split method.

Datasets	Classifiers	70:30				80:20				90:10			
		TPR	FNR	TNR	FPR	TPR	FNR	TNR	FPR	TPR	FNR	TNR	FPR
Dataset_1	RF	1.0	0.0	1.0	0.0	1.0	0.0	1.0	0.0	1.0	0.0	1.0	0.0
	GB	0.987	0.012	1.0	0.0	0.980	0.019	1.0	0.0	0.961	0.038	1.0	0.0
	AdaB	1.0	0.0	1.0	0.0	0.980	0.019	1.0	0.0	1.0	0.0	1.0	0.0
	NB	0.948	0.051	0.954	0.045	0.980	0.019	0.964	0.035	0.961	0.038	0.928	0.071
	SVM	0.974	0.025	0.977	0.022	1.0	0.0	0.964	0.035	1.0	0.0	0.928	0.071
	XGB	0.987	0.012	1.0	0.0	0.980	0.019	1.0	0.0	0.961	0.038	1.0	0.0
	DT	0.974	0.025	1.0	0.0	0.980	0.019	1.0	0.0	1.0	0.0	1.0	0.0
Dataset_2	RF	0.987	0.012	1.0	0.0	1.0	0.0	1.0	0.0	1.0	0.0	1.0	0.0
	GB	1.0	0.0	1.0	0.0	1.0	0.0	1.0	0.0	1.0	0.0	1.0	0.0
	AdaB	0.987	0.012	1.0	0.0	0.980	0.019	1.0	0.0	0.961	0.038	1.0	0.0
	NB	0.935	0.064	0.954	0.045	0.961	0.038	0.964	0.035	0.961	0.038	0.928	0.071
	SVM	0.935	0.064	1.0	0.0	0.961	0.038	1.0	0.0	0.961	0.038	1.0	0.0
	XGB	1.0	0.0	1.0	0.0	1.0	0.0	1.0	0.0	1.0	0.0	1.0	0.0
	DT	0.974	0.025	1.0	0.0	0.961	0.038	1.0	0.0	0.961	0.038	1.0	0.0
Dataset_3	RF	0.883	0.116	1.0	0.0	0.923	0.076	1.0	0.0	0.961	0.038	1.0	0.0
	GB	0.883	0.116	1.0	0.0	0.923	0.076	1.0	0.0	0.961	0.038	1.0	0.0
	AdaB	0.883	0.116	1.0	0.0	0.923	0.076	1.0	0.0	0.961	0.038	1.0	0.0
	NB	0.883	0.116	0.954	0.045	0.923	0.076	0.964	0.035	0.961	0.038	1.0	0.0
	SVM	0.883	0.116	1.0	0.0	0.923	0.076	1.0	0.0	0.961	0.038	1.0	0.0
	XGB	0.870	0.129	0.909	0.090	0.942	0.057	0.892	0.107	0.961	0.038	1.0	0.0
	DT	0.883	0.116	1.0	0.0	0.923	0.076	1.0	0.0	0.961	0.038	1.0	0.0
Dataset_4	RF	1.0	0.0	1.0	0.0	1.0	0.0	1.0	0.0	1.0	0.0	1.0	0.0
	GB	0.987	0.012	0.977	0.022	0.980	0.019	1.0	0.0	0.961	0.038	0.928	0.071
	AdaB	1.0	0.0	1.0	0.0	1.0	0.0	1.0	0.0	1.0	0.0	1.0	0.0
	NB	0.948	0.051	0.954	0.045	0.980	0.019	0.964	0.035	0.961	0.038	0.928	0.071
	SVM	0.948	0.051	1.0	0.0	1.0	0.0	1.0	0.0	1.0	0.0	1.0	0.0
	XGB	0.987	0.012	1.0	0.0	0.980	0.019	1.0	0.0	0.961	0.038	1.0	0.0
	DT	0.987	0.012	1.0	0.0	0.980	0.019	1.0	0.0	1.0	0.0	1.0	0.0
Dataset_5	RF	1.0	0.0	1.0	0.0	1.000	0.000	1.0	0.0	1.0	0.0	1.0	0.000
	GB	0.987	0.012	1.0	0.0	0.980	0.019	1.0	0.0	0.961	0.038	1.0	0.000
	AdaB	1.0	0.0	1.0	0.0	0.980	0.019	1.0	0.0	1.0	0.0	1.0	0.000
	NB	0.935	0.064	0.954	0.045	0.961	0.038	0.964	0.035	1.0	0.0	0.928	0.071
	SVM	0.987	0.012	1.0	0.0	1.000	0.000	1.0	0.000	1.0	0.0	1.000	0.000
	XGB	0.987	0.012	0.977	0.022	0.980	0.019	0.964	0.035	0.961	0.038	0.928	0.071
	DT	0.987	0.012	1.0	0.0	0.980	0.019	1.0	0.0	1.0	0.0	0.928	0.071
Dataset_6	RF	0.987	0.012	0.977	0.022	0.980	0.019	0.964	0.035	0.961	0.038	1.0	0.0
	GB	0.987	0.012	0.977	0.022	1.0	0.0	0.964	0.035	0.961	0.038	1.0	0.0
	AdaB	0.974	0.025	1.0	0.0	0.980	0.019	0.964	0.035	0.961	0.038	1.0	0.0
	NB	0.935	0.064	0.954	0.045	0.980	0.019	0.964	0.035	0.961	0.038	1.0	0.0
	SVM	0.909	0.090	1.0	0.0	0.942	0.057	1.0	0.0	0.961	0.038	1.0	0.0
	XGB	0.974	0.025	0.977	0.022	0.980	0.019	0.964	0.035	0.961	0.038	1.0	0.0
	DT	0.961	0.038	0.931	0.068	0.942	0.057	0.964	0.035	0.923	0.076	1.0	0.0
Dataset_7	RF	0.935	0.064	0.977	0.022	0.923	0.076	0.964	0.035	0.961	0.038	1.0	0.0
	GB	0.922	0.077	0.954	0.045	0.903	0.096	0.964	0.035	0.923	0.076	1.0	0.0
	AdaB	0.974	0.025	0.954	0.045	0.961	0.038	1.0	0.0	0.923	0.076	1.0	0.0
	NB	0.935	0.064	0.909	0.090	0.980	0.019	0.892	0.107	0.961	0.038	0.928	0.071
	SVM	0.909	0.090	1.0	0.0	0.961	0.038	1.0	0.0	0.961	0.038	1.0	0.0
	XGB	0.935	0.064	0.954	0.045	0.923	0.076	0.928	0.071	0.961	0.038	1.0	0.0
	DT	0.935	0.064	0.931	0.068	0.961	0.038	0.964	0.035	0.923	0.076	1.0	0.0

Table 8 delineates an in-depth analysis of classifiers, focusing on TPR, FNR, TNR, and FPR metrics across various datasets and split ratios. RF and AdaB emerge as top performers, boasting high TPR and low FPR across 70:30, 80:20, and 90:10 splits, underscoring their effectiveness in kidney disease prediction. In contrast, NB exhibits variability, performing well on some datasets but facing challenges with complex feature interactions, reflected in inconsistent FPR values. XgB, distinguished by its regularization and parallel processing features, often displays a marginal upper hand, marking it as a strong contender for practical applications.

Table 9 unveils the AUC values for different classifiers across three different train-test split ratios (70:30, 80:20, 90:10) for seven different datasets. For the 70:30 split, RF and AdaB classifiers achieve perfect AUC scores of 1.0 on Datasets 1, 2, 4, and 5, with GB also obtaining a score of 1.0 on Datasets 2 and 5. In the 80:20 split, RF and AdaB continue their strong performance with perfect scores on the same datasets, while SVM and GB achieve a score of 1.0 on Datasets 4 and 5. With the 90:10 split, AdaB and RF again show perfect scores on Datasets 1, 2, 4, and 5, and DT reaches an AUC of 1.0 on Datasets 1 and 4. Overall, RF and AdaB classifiers demonstrate the highest effectiveness across various datasets and split ratios, with GB also showing robust results. This suggests that these classifiers are likely the best choices for the datasets analyzed when considering AUC as the performance metric.

**Table 9:** AUC values of different classifiers for different splitting ratios based on train-test split method.

Classifiers	Split Ratio	AUC						
		Dataset_1	Dataset_2	Dataset_3	Dataset_4	Dataset_5	Dataset_6	Dataset_7
RF	70:30	1.0	1.0	0.943	1.0	1.0	0.999	0.997
GB	70:30	0.999	1.0	0.943	0.999	1.0	0.998	0.992
AdaB	70:30	1.00	1.0	0.943	1.0	1.0	0.999	0.997
NB	70:30	0.973	0.969	0.919	0.973	0.972	0.972	0.969
SVM	70:30	0.998	0.994	0.939	1.0	1.0	0.987	0.987
XGB	70:30	1.0	1.0	0.938	0.999	0.999	0.999	0.986
DT	70:30	0.987	1.0	0.943	0.993	0.993	0.946	0.933
RF	80:20	1.0	1.0	0.967	1.0	1.0	0.998	0.995
GB	80:20	0.999	1.0	0.967	1.0	1.0	0.997	0.989
AdaB	80:20	1.0	1.0	0.967	1.0	1.0	0.999	0.995
NB	80:20	0.980	0.978	0.948	0.980	0.978	0.980	0.979
SVM	80:20	0.999	0.997	0.965	1.0	1.0	0.996	0.995
XGB	80:20	0.999	1.0	0.967	0.999	0.999	0.998	0.986
DT	80:20	0.990	1.0	0.967	0.990	0.990	0.953	0.962
RF	90:10	1.0	1.0	0.979	1.0	1.0	0.997	0.997
GB	90:10	0.997	1.0	0.979	0.997	1.0	1.000	1.000
AdaB	90:10	1.0	1.0	0.979	1.0	1.0	1.000	0.989
NB	90:10	0.997	0.997	0.979	0.997	0.997	0.997	0.997
SVM	90:10	1.0	0.997	0.979	1.0	1.0	0.997	0.997
XGB	90:10	0.997	1.0	0.979	0.997	0.997	1.000	1.0
DT	90:10	1.0	1.0	0.979	1.0	0.964	0.961	0.961

The 70:30 split ratio sees the best performance, with RF and AdaB achieving perfect scores in several datasets. This can be attributed to the balanced distribution of data, allowing for effective training and validation. The high AUC scores indicate these classifiers' ability to generalize well, making them suitable for these datasets. Performance dips are observed with the 90:10 split, where the smaller testing set may not capture the full data complexity, leading to potential overfitting issues. The high training data proportion could result in models that are too tailored to the training set, reducing their effectiveness on unseen data. Overall, RF and AdaB consistently outperform across various datasets and splits, with GB also showing strong results. These classifiers, particularly in the 70:30 split, demonstrate robustness and generalization capabilities, making them preferred choices for these datasets.

From Table 10**,** it is observed that NB consistently shows the lowest training and testing times across all datasets and split ratios, indicating its efficiency in terms of computational cost. Conversely, GB and RF generally have higher training times, which can be attributed to the complexity of these ensemble methods. For the 70:30 split ratio, AdaB tends to have lower training times than GB and RF on most datasets, while maintaining competitive testing times. SVM and DT also demonstrate relatively low training and testing times across various datasets. As the splitting ratio shifts towards more training data (80:20 and 90:10), there's an increase in training times for all classifiers, which is expected due to the larger volume of data being processed. However, the increase is not uniform across classifiers. For instance, RF and GB show a more significant rise in training times compared to AdaB, NB, SVM, XGB, and DT. The summary of this analysis underscores that Naive Bayes is the most time-efficient classifier across all scenarios, while more complex models like RF and GB require more computational time for training. AdaB strikes a balance with relatively lower training times and efficient testing times, making it a suitable candidate for scenarios where a trade-off between time complexity and classifier performance is critical. DT and SVM also offer good time efficiency, which may make them preferable in time-sensitive applications.

**Table 10:** Time complexity analysis of for different classifiers for different splitting ratios based on train-test split method.

Dataset name	Classifiers	70:30		80:20		90:10	
		Training time (s)	Testing time (s)	Training time (s)	Testing time (s)	Training time (s)	Testing time (s)
Dataset_1	RF	0.294	0.024	0.396	0.025	0.385	0.027
	GB	0.347	0.002	0.416	0.001	0.427	0.001
	AdaB	0.226	0.032	0.184	0.027	0.163	0.015
	NB	0.006	0.001	0.005	0.002	0.001	0.001
	SVM	0.017	0.002	0.027	0.006	0.017	0.002
	XGB	0.091	0.005	0.101	0.006	0.088	0.005
	DT	0.006	0.002	0.008	0.005	0.010	0.002
Dataset_2	RF	0.407	0.018	0.247	0.017	0.261	0.015
	GB	0.195	0.002	0.154	0.001	0.150	0.001
	AdaB	0.162	0.019	0.125	0.015	0.121	0.015
	NB	0.002	0.001	0.001	0.000	0.001	0.001
	SVM	0.017	0.002	0.012	0.002	0.014	0.001
	XGB	0.080	0.006	0.074	0.004	0.075	0.004
	DT	0.005	0.002	0.005	0.003	0.006	0.002
Dataset_3	RF	0.294	0.020	0.371	0.035	0.332	0.031
	GB	0.186	0.003	0.277	0.001	0.148	0.001
	AdaB	0.172539	0.025	0.195	0.029	0.159	0.023
	NB	0.003990	0.002	0.006	0.005	0.003	0.000
	SVM	0.017952	0.002	0.020	0.002	0.017	0.001
	XGB	0.060835	0.056	0.085	0.004	0.063	0.004
	DT	0.003990	0.001	0.005	0.002	0.005	0.001
Dataset_4	RF	0.341350	0.024	0.373	0.026	0.335	0.025
	GB	0.420877	0.002	0.411	0.001	0.456	0.002
	AdaB	0.242354	0.021	0.198	0.017	0.212	0.021
	NB	0.003	0.002	0.002	0.002	0.003	0.000
	SVM	0.016	0.006	0.018	0.003	0.024	0.004
	XGB	0.084	0.004	0.078	0.003	0.085	0.005
	DT	0.007	0.002	0.008	0.001	0.007	0.002
Dataset_5	RF	0.328	0.025	0.325	0.028	0.262	0.016
	GB	0.289	0.002	0.277	0.003	0.266	0.000
	AdaB	0.185	0.020	0.172	0.024	0.152	0.014
	NB	0.002	0.001	0.002	0.001	0.002	0.001
	SVM	0.015	0.003	0.017	0.001	0.014	0.001
	XGB	0.070	0.004	0.058	0.004	0.066	0.004
	DT	0.005	0.002	0.005	0.001	0.006	0.002
Dataset_6	RF	0.329	0.023	0.433	0.022	0.490	0.064
	GB	0.355	0.003	0.460	0.002	0.435	0.001
	AdaB	0.277	0.029	0.264	0.030	0.232	0.023
	NB	0.004	0.002	0.004	0.002	0.004	0.000
	SVM	0.029	0.002	0.031	0.005	0.036	0.006
	XGB	0.090	0.005	0.085	0.005	0.092	0.006
	DT	0.006	0.002	0.007	0.002	0.010	0.002
Dataset_7	RF	0.290	0.023	0.356	0.027	0.382	0.026
	GB	0.299	0.001	0.304	0.002	0.327	0.002
	AdaB	0.207	0.022	0.233	0.048	0.228	0.019
	NB	0.004	0.001	0.003	0.002	0.002	0.001
	SVM	0.023	0.002	0.023	0.002	0.024	0.002
	XGB	0.097	0.006	0.090	0.004	0.089	0.003
	DT	0.007	0.002	0.006	0.002	0.007	0.002

To assess the efficiency of our model, we conducted a comparison with the results of models proposed by other authors, as presented in Table 11.

**Table 11:** Comparison with state-of-the-art works using same dataset.

Authors	Split ratio	Model name	Accuracy
M.A. Islam et al. (2023)^8^	70:30	XgBoost	98.3 %
R. Sawhney et al. (2023)^9^	70:30	ANN	100%
Alsekait D.M. et al. (2023)^6^	80:20	DL with SVM	99.69
Arif M.S. et al. (2023)^10^	80:20	KNN	100
Poonia RC et al. (2022)^11^	80:20	LR	98.75
Pal S. (2022)^12^	80:20	DT	97.23
K.M. Almustafa (2021)^13^	K-Fold=10	J48	99.75
Ilyas et al. (2021)^14^	K-Fold=15	J48	85.5%
Our study	70:30, 80:20, K-Fold=10, 15	RF, AdaB	100%

The analysis spans studies from 2021 to 2023, showcasing a variety of data split ratios including 70:30, 80:20, and K-Fold validation with either 10 or 15 folds used by the different authors. The comparative study encompasses a broad spectrum of models such as XgB, ANN, DL with SVM, KNN, LR, DT, and J48, with the recorded accuracies ranging from 85.5% to the highest mark of 100%. The proposed model in the document, which employs a synergy of Random Forest and AdaBoost (RF AdaB), stands out by achieving a perfect accuracy of 100%, showcasing its superior performance. This is observed across various validation methods, with split ratios of 70:30, 80:20, and K-Fold set to 10, and 15, indicating the robustness and efficacy of the proposed model in comparison to its contemporaries.

## Web application development

**Fig. 4:**
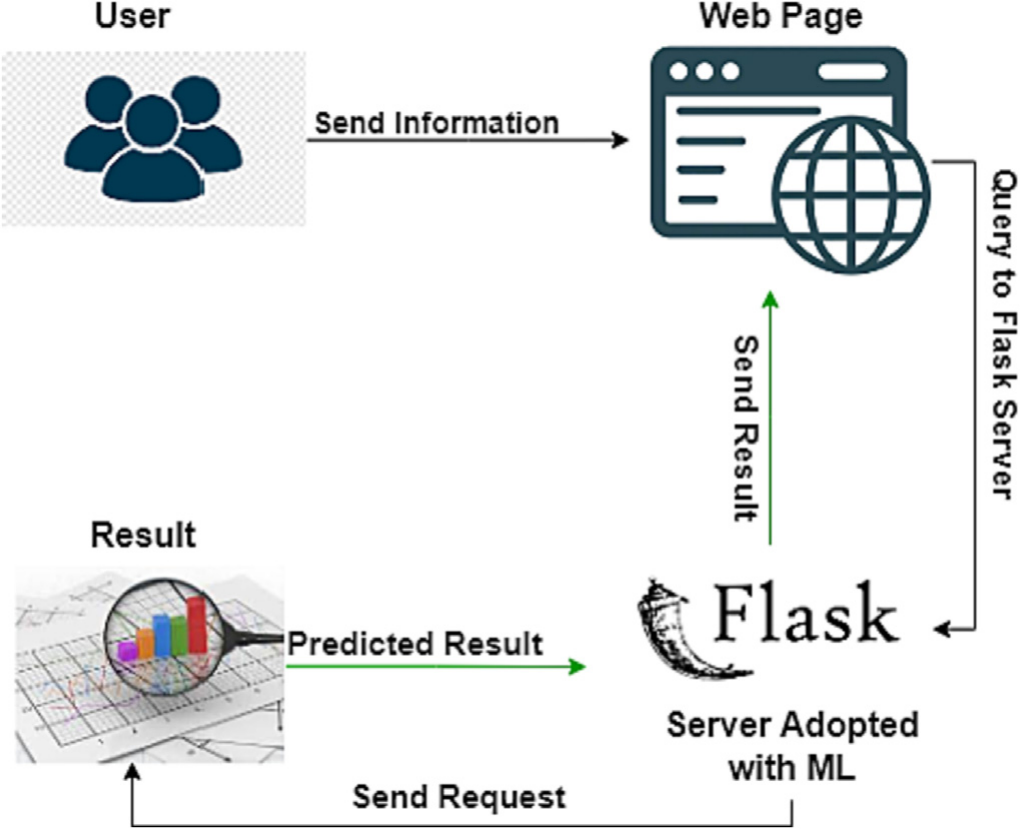
Working process of CKD web app.

Detecting CKD is crucial in contemporary research. Medical science offers advanced but expensive treatments for CKD testing, making it difficult for the average person to afford them. A web application for CKD detection could simplify this process. In this research paper, we introduce a machine learning-based, web application developed using Flask. Flask is a simple Python framework for creating web applications. This user-friendly system prompts users to input specific information; it then evaluates whether they have CKD. A “Positive” or “Negative” result is displayed on the web portal, and users can print the report for additional consultation. Fig. 4 illustrates the application’s basic file structure.

**Fig. 5:**
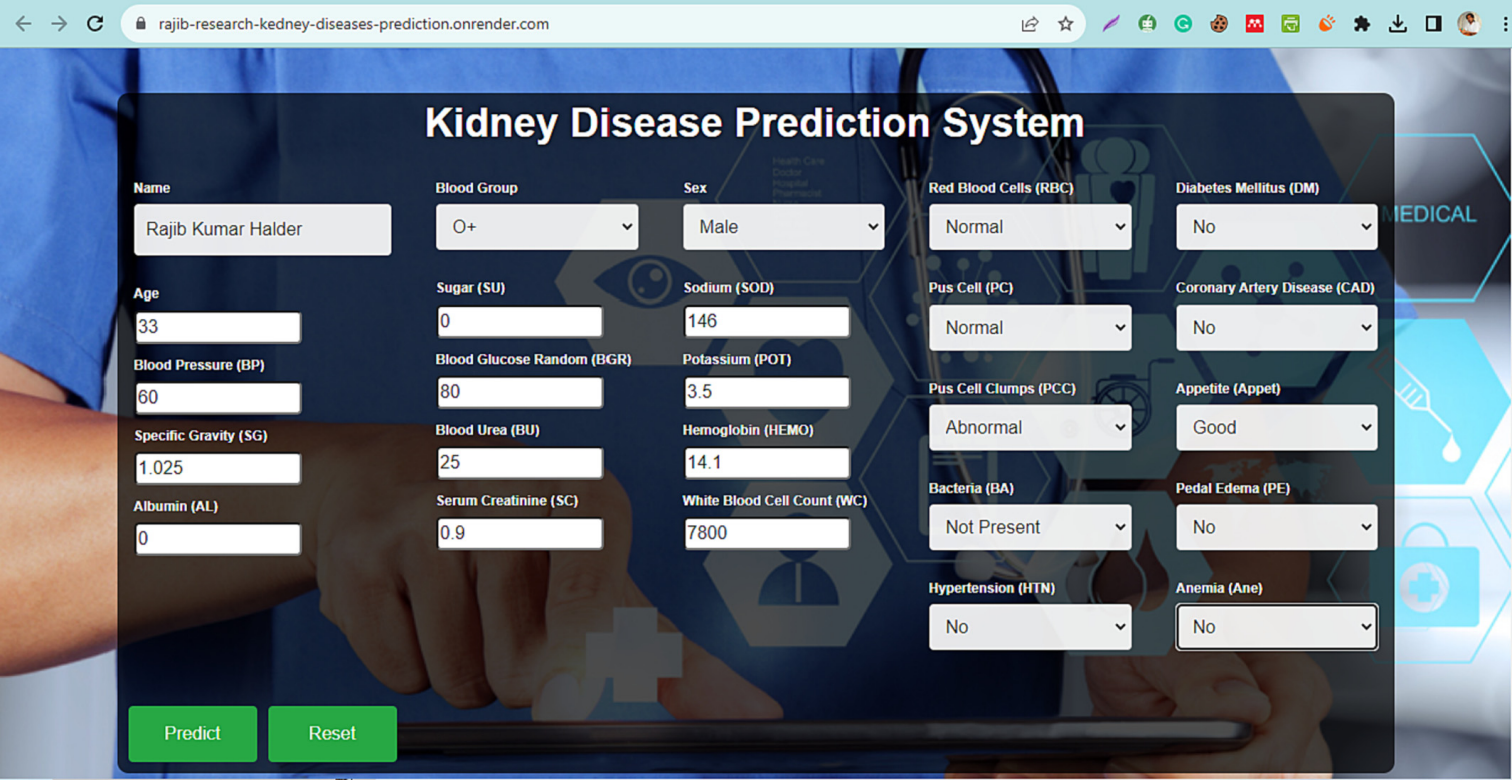
Input form.

**Fig. 6:**
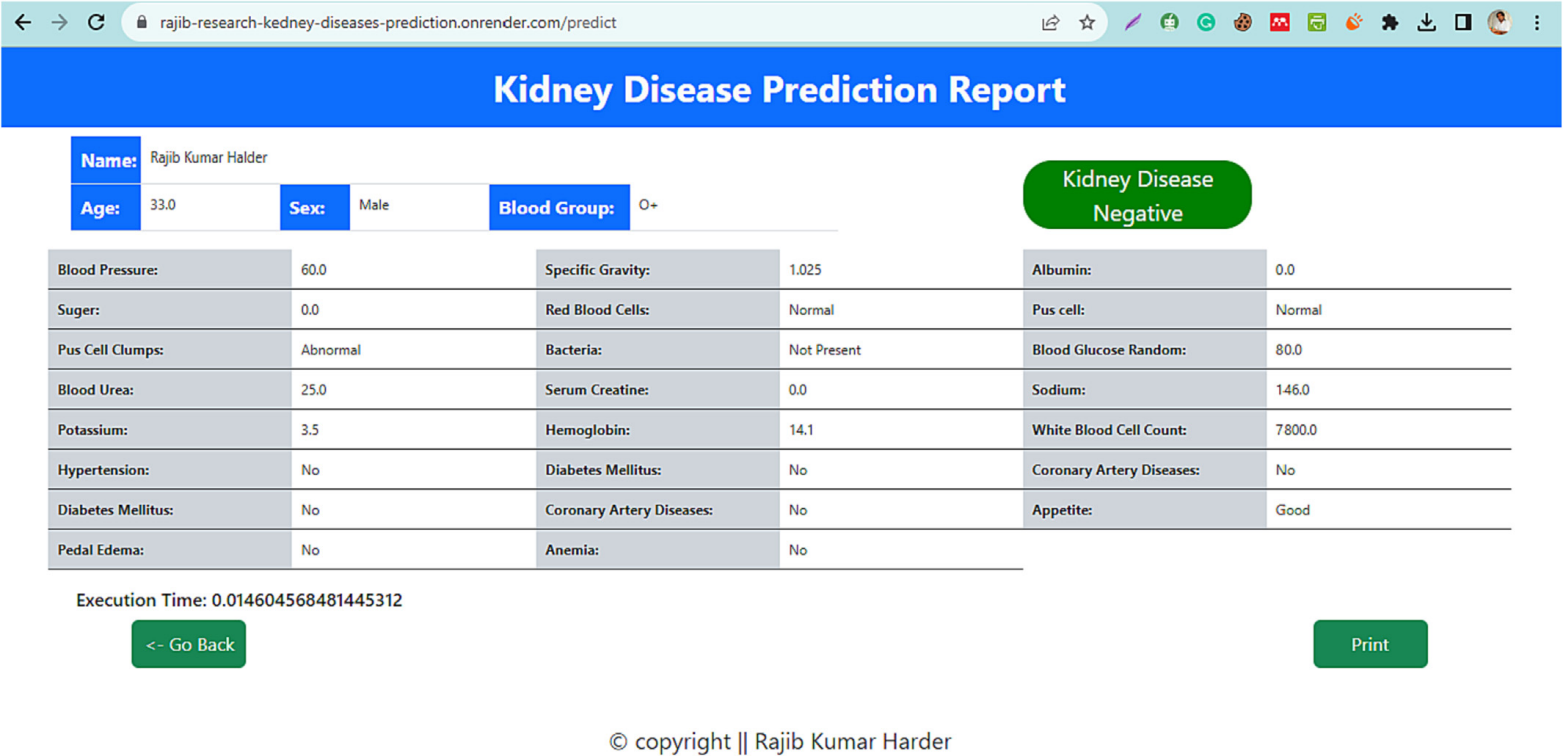
Result page.

**Fig. 7:**
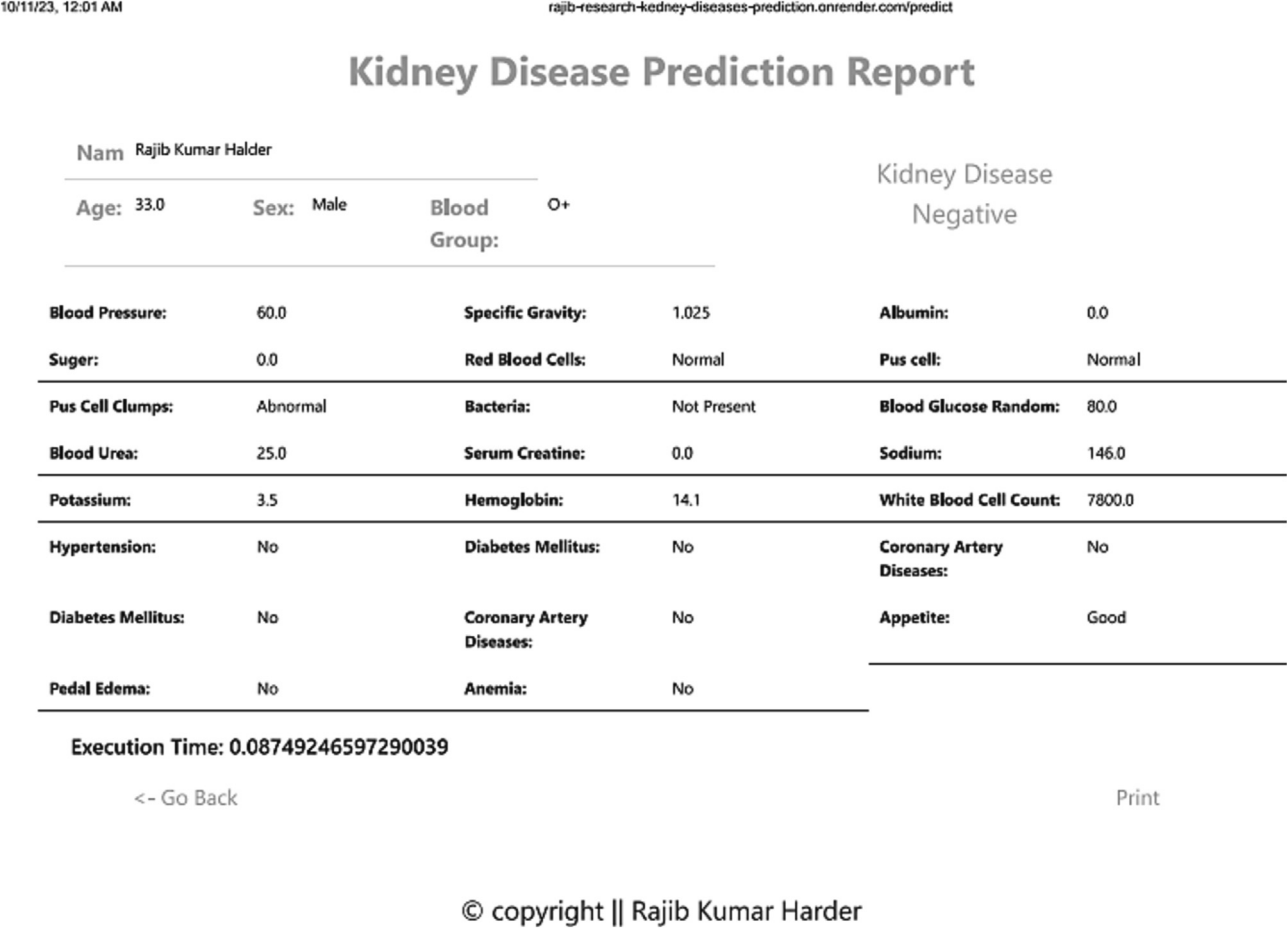
Report pdf file.

The development process involves four program modules. The ‘model.pkl’ file houses the machine learning model for CKD prediction, incorporating the most accurate model. The ‘app.py’ package contains Flask APIs that gather information about kidney disease from the user, compute predictions with the model, and return the results. Users can input CKD-related information on the ‘index.html’ page and view the predicted results on the ‘result.html’ page. Users submit the required information via the homepage, which is then processed by the backend. A Flask server, equipped with the machine learning algorithm, generates the predictions, displayed in the “Results” section. Datasets 1 and 4 consistently exhibited the most commendable performance across various classifiers, notably RF and AdaB, achieving levels of accuracy that were either perfect or in close proximity to perfection. This observation hints at the possibility that the features within these datasets are well-structured and discernible by the classifiers, or that the datasets themselves are devoid of intricate or noisy data. Consequently, we have made the decision to employ the RF classifier from the available options and opt for Dataset_1 as the training data for our forthcoming flagship application. Fig. 5, Fig. 6, Fig. 7 showcases screenshots of various web pages. Our Flask app is hosted on Render, a unified cloud service that supports the building and running of apps and websites, offering features like free TLS certificates, a global CDN, private networks, and automatic deploys from Git. The live application is accessible at the following link: https://rajib-research-kedney-diseases-prediction.onrender.com/.

Fig. 5 includes a mix of HTML, CSS, and a small portion of JavaScript. Here's an overview of its structure and functionality:

HTML structure: The file starts with the standard DOCTYPE html declaration, followed by the html element with a specified language attribute (lang=“en”). The head section includes meta tags for character set (UTF-8) and viewport settings, a link to Bootstrap 5.3.2 for styling, and an embedded style section for custom CSS.

Styling (CSS): The CSS styles are extensive, defining the appearance of body, containers, headings, labels, inputs, forms, and buttons. It includes responsive design media queries to adjust layout for different screen sizes. Key features include a background image, font settings, grid layout for form elements, button styles, and hover effects.

Web page content: The body contains a div with a class container, which includes a heading and a form. The form is for predicting kidney disease and has various input fields like name, blood group, sex, age, blood pressure, specific gravity, albumin, sugar, blood glucose random, blood urea, serum creatinine, sodium, potassium, hemoglobin, white blood cell count, red blood cells, pus cell, pus cell clumps, bacteria, hypertension, diabetes mellitus, coronary artery disease, appetite, pedal edema, anemia related to medical parameters. It uses both text inputs and select dropdowns. The form structure is divided into columns for layout organization.

Form action: The form is set to post data to a/predict endpoint, indicating that it's likely intended to interact with a backend server for processing the input data. Additionally, there's an action attribute set to “./result.html” which might be a fallback or an error in the code since having two action attributes is unusual.

JavaScript: Two JavaScript functions (main() and myfun()) are referenced in the submit and reset buttons, but their definitions are not included in the document. These are likely defined elsewhere, possibly for handling the form submission and resetting form data.

Fig. 6 is a well-structured HTML file designed for presenting a Kidney Disease Prediction Report. It uses HTML for structure, CSS for styling, and likely some JavaScript for functionality (though the JavaScript code isn't included in this file). Key features include:

HTML structure: Standard HTML5 document structure with a 'DOCTYPE' declaration and language set to English.

Bootstrap integration: Uses Bootstrap 5.3.2 for responsive design and pre-defined styles.

Custom CSS: The CSS provides specific styles for elements like the result box, headings, and buttons, including font sizes, colors, and layout adjustments.

Content layout: The body includes a section for the report title and a div for the main content. There's a table displaying patient information and prediction results, formatted with Bootstrap classes.

Dynamic data: The FILE uses placeholders (like {{ name }}, {{ age }}, etc.) for dynamic data insertion, suggesting integration with a backend or JavaScript for data rendering.

User interaction: Elements for user interaction are present, such as buttons for going back and printing the report.

## Findings

The research presented in machine learning-based kidney diseases prediction (ML-CKDP) has significant implications for medical diagnostics and the accessibility of healthcare services. It introduces a machine learning-based web application developed with Flask that provides an affordable and accessible approach for CKD testing, which is crucial given the high costs associated with medical treatments for CKD. Users can input health data and receive immediate predictions on their CKD status, simplifying the diagnostic process and making it more accessible. In Fig. 3, Datasets_1 consistently showed the best performance. It employs a filter method based on correlation analysis for feature selection, identifying crucial predictors like age, blood pressure, specific gravity, albumin, sugar, blood glucose random, blood urea, serum creatinine, sodium, potassium, hemoglobin, white blood cell count, red blood cells, pus cell, pus cell clumps, bacteria, hypertension, diabetes mellitus, coronary artery disease, appetite, pedal edema, and anemia. The application's efficacy is highlighted by the high accuracy of classifiers like RF and AdaB in certain dataset, suggesting well-defined and balanced features. This approach not only simplifies CKD diagnosis but also enhances healthcare decision-making with accurate predictions, indicating a significant advancement in applying machine learning to health care.

The methodology has notable limitations. First, using label encoding for categorical variables can be problematic for nominal data, as it may lead to incorrect assumptions about the data's ordinal nature. Second, there's a significant risk of overfitting with complex models like RF and XgBoost, which requires careful regularization and tuning. Finally, the effectiveness of this approach varies across different datasets and problem domains, suggesting a need for tailored strategies depending on the specific characteristics of each dataset. Recognizing these limitations of our proposed model, we suggest potential avenues to address these challenges:

1. To mitigate the issue of label encoding for categorical variables, especially for nominal data, alternative encoding methods such as one-hot encoding or embedding layers can be employed. These methods prevent the model from making incorrect assumptions about the ordinal nature of the data.
2. The risk of overfitting in complex models like RF and XgBoost can be addressed by implementing more rigorous regularization and hyperparameter tuning strategies. Utilizing techniques such as cross-validation and grid search can help in finding the optimal model configuration that generalizes better to unseen data.
3. Given the varying effectiveness of our approach across different datasets and problem domains, it is advisable to develop tailored strategies for each dataset. This involves conducting thorough exploratory data analysis to understand dataset-specific characteristics and adapting the modeling approach accordingly. Customizing feature selection, model architecture, and hyperparameters based on the unique aspects of each dataset can lead to more robust and effective models.

## Conclusion

Our study marks a significant advancement in the field of predictive diagnostics for CKDs, primarily through the development of a sophisticated machine learning model and a user-centric web application. Addressing the urgent need for early and accurate detection of CKD, particularly critical in low- and middle-income countries due to the disease's growing global impact, our approach set itself apart by employing a comprehensive preprocessing protocol and a variety of feature selection techniques. These methods significantly enhanced the robustness and accuracy of our CKD classification model. The implications of our research, as detailed in ML-CKDP, are profound in the realms of medical diagnostics and healthcare accessibility. We introduced a machine learning-enabled web application, built with Flask, which offers an affordable and accessible approach to CKD testing. This innovation is particularly important considering the high costs associated with CKD medical treatments. The application enables users to input their health data and receive immediate predictions regarding their CKD status, thus simplifying the diagnostic process and making it more accessible to a broader audience. Our findings, as illustrated in Fig. 3, demonstrate that Datasets_1 exhibited the best performance, utilizing a filter method based on correlation analysis for feature selection and identifying crucial predictors like age, blood pressure, specific gravity, albumin, sugar, blood glucose random, blood urea, serum creatinine, sodium, potassium, hemoglobin, white blood cell count, red blood cells, pus cell, pus cell clumps, bacteria, hypertension, diabetes mellitus, coronary artery disease, appetite, pedal edema, anemia. The application's efficacy is further highlighted by the high accuracy of classifiers such as RF and AdaB in certain datasets, suggesting well-defined and balanced features. This approach not only simplifies the process of CKD diagnosis but also enhances healthcare decision-making through accurate predictions, indicating significant progress in the application of machine learning in health care. The significant contributions of our research are multifaceted. We developed a machine learning classification model that utilized a range of classifiers, including RF, AdaB, GB, XgB, NB, SVM, and DT, applied to preprocessed datasets to determine the most effective model. We also employed a broad suite of feature selection methods, from heatmap correlation analysis and chi-square tests to variance thresholds, recursive feature elimination, sequential forward selection, lasso, and ridge regression. These techniques were instrumental in identifying and retaining influential features, facilitating a detailed investigation of feature influence. Additionally, we prepared seven distinct datasets, each meticulously preprocessed using varied feature selection methods, enabling an exhaustive evaluation of different feature sets and their effects on classification accuracy. Our performance evaluation involved a comparative analysis against state-of-the-art works, using various train-test splits and stratified k-fold cross-validation with multiple folds. This ensured a comprehensive and robust assessment of the classifiers' performance across different scenarios. We also conducted an in-depth investigation into the time complexity of our deployed methodology, offering valuable insights for practical application decisions. Beyond these methodological advancements, we crafted a user-oriented web application that facilitates the entry of kidney health parameters and associated risk factors. This tool, leveraging our refined datasets and classifiers, delivers instant predictions and risk evaluations for kidney-related ailments, exemplifying the practical application of our research and encouraging preemptive health management.

## Declaration of competing interest

The authors declare that they have no conflict of interest and no known competing financial interests or personal relationships that could have appeared to influence the work reported in this paper.

## Data Availability

All datasets and code are available at the link below: 1. Datasets and Source Code: https://github.com/rajib1346/MLCkd-Source_Code.git. 2. Web app source code: https://github.com/rajib1346/MLCkd-Web-App.git.
